# Cerebellum and neurodevelopmental disorders: RORα is a unifying force

**DOI:** 10.3389/fncel.2023.1108339

**Published:** 2023-03-30

**Authors:** Svethna Ribeiro, Rachel M. Sherrard

**Affiliations:** UMR 8256 Biological Adaptation and Ageing, Sorbonne Université and CNRS, IBPS-B2A, Paris, France

**Keywords:** cerebellum, autism, schizophrenia, Purkinje cell, circadian rhythms

## Abstract

Errors of cerebellar development are increasingly acknowledged as risk factors for neuro-developmental disorders (NDDs), such as attention deficit hyperactivity disorder (ADHD), autism spectrum disorder (ASD), and schizophrenia. Evidence has been assembled from cerebellar abnormalities in autistic patients, as well as a range of genetic mutations identified in human patients that affect the cerebellar circuit, particularly Purkinje cells, and are associated with deficits of motor function, learning and social behavior; traits that are commonly associated with autism and schizophrenia. However, NDDs, such as ASD and schizophrenia, also include systemic abnormalities, e.g., chronic inflammation, abnormal circadian rhythms etc., which cannot be explained by lesions that only affect the cerebellum. Here we bring together phenotypic, circuit and structural evidence supporting the contribution of cerebellar dysfunction in NDDs and propose that the transcription factor Retinoid-related Orphan Receptor alpha (RORα) provides the missing link underlying both cerebellar and systemic abnormalities observed in NDDs. We present the role of RORα in cerebellar development and how the abnormalities that occur due to RORα deficiency could explain NDD symptoms. We then focus on how RORα is linked to NDDs, particularly ASD and schizophrenia, and how its diverse extra-cerebral actions can explain the systemic components of these diseases. Finally, we discuss how RORα-deficiency is likely a driving force for NDDs through its induction of cerebellar developmental defects, which in turn affect downstream targets, and its regulation of extracerebral systems, such as inflammation, circadian rhythms, and sexual dimorphism.

## 1. Introduction

Neurodevelopmental disorders (NDDs) include a wide range of dysfunction such as autism spectrum disorder (ASD), schizophrenia, attention deficit hyperactivity disorder (ADHD), dyslexia etc. While each disorder is characterized by a well-defined set of symptoms described in the Diagnosis and Statistical Manual of Mental Disorders (DSM-5 TR), there is also a large overlap in symptomatology, such as learning difficulties and diminished social interaction, as well as genetic abnormalities (Carroll and Owen, 2009).

**FIGURE 1:**
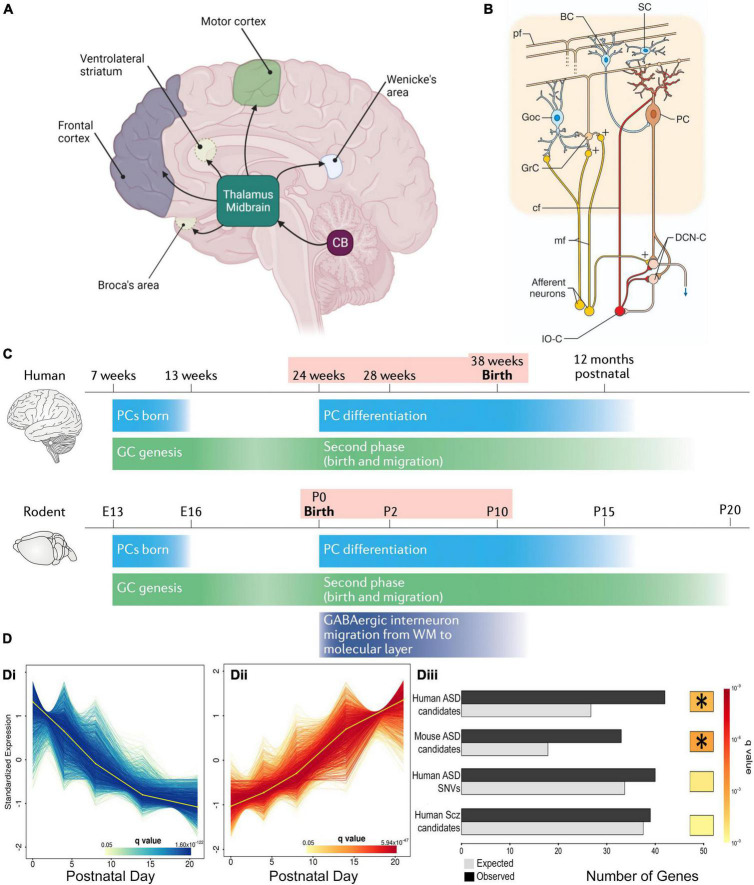
Cerebellar connectivity and development underlies its link to NDDs. **(A)** The cerebellum has extensive connectivity with the forebrain *via* the thalamus which underlie its many cognitive and affective roles that are concentrated in different regions. Reproduced with permission from Hatten (2020). **(B)** Cerebellar cortical circuitry, in which Purkinje cells (PCs) integrate signals from olivary climbing fibers (cf) and granule cell (GrC) parallel fibers (pf), which have received cortical information *via* pontine mossy fibers (mf), in order to modify deep cerebellar nuclear (DCN) activity, which in turn projects back to the cortex. Neuronal responses are modulated by GABAergic interneurons: feedforward inhibition through basket (BC) and stellate (SC) cells to PCs, and feedback inhibition of GrC-pf activity by Golgi cells (GoC). Reproduced from D’Angelo and Casali (2013) with permission. **(C)** Humans and rodents have a relatively similar time-course of events leading to cerebellar development, although the human brain inevitably takes longer. Both human and rodent cerebella have relatively similar prolonged vulnerability (pink shading) around birth. Reproduced with permission from Sathyanesan et al. (2019). **(D)** DESeq2 analysis of mouse PC RNA differentially expressed during early post-natal development **(Di, Dii)**. The gene group that increases expression with development **(Dii)** was significantly enriched with ASD risk candidate genes **(Diii)** but, interestingly, not schizophrenia risk genes. *Indicates a significant difference. Modified from Clifford et al. (2019).

Of all the different brain components involved in NDDs, the cerebellum was proposed as a key region because NDD patients often present with multiple sensory-motor integration deficits that are symptomatic of altered cerebellar function (Fatemi et al., 2012). Also the cerebellum’s broad connectivity indicates its involvement in sensory-motor, cognitive and affective processing (Figure 1), wherein cerebellar integration is necessary for their correct organization (Glickstein and Doron, 2008; Strick et al., 2009; Buckner, 2013; Koziol et al., 2014; D’Mello and Stoodley, 2015). Thus, we can apply the theory of cognitive dysmetria, originally applied to cerebellar neurodegenerative disease (Schmahmann, 1998), to understand the pertinence of the cerebellum to the dysfunctions observed in NDDs.

However, cerebellar behavioral dysfunction will also involve many non-cerebellar areas, and is therefore only indirect support for the cerebellar-NDD hypothesis. Also, it does not identify whether the cerebellar deficit generates the NDD or is merely a reflection of disordered brain development. Support for a causal role for the cerebellum comes from it having the highest co-expression of NDD-associated genes (Menashe et al., 2013; Wang et al., 2014), many of which are temporally regulated in developing Purkinje neurons (Clifford et al., 2019), and are thus central to cerebellar development (Parenti et al., 2020). Conversely, cerebellar dysfunction alone is not sufficient to explain the systemic comorbidities often seen in NDDs, such as abnormal circadian rhythm, chronic inflammation and sexual dimorphism (Hu et al., 2015; Siniscalco et al., 2018).

Here we propose a potential unifying theory in which a pleiotropic nuclear receptor, Retinoic acid-related Orphan Receptor alpha (RORα), could account for the cerebellar, neuropsychiatric and systemic components of neurodevelopmental disorders. Of the 1,353 mouse genes that have been linked to NDDs (SFARI Gene 3.0, 2022) 14% are likely *RORA* targets (Hu et al., 2015). Moreover, we chose RORα because in addition to its regulation of critical stages of cerebellar development and function, it also has generic physiological roles in many systems including inflammation, circadian rhythms and sex steroid metabolism (Jarvis et al., 2002; Boukhtouche et al., 2006b; Hu et al., 2015), processes that also affected in NDDs. Moreover, while the cerebellum-NDD hypothesis has been extensively reviewed (Wang et al., 2014; Sathyanesan et al., 2019; Thabault et al., 2022), including its potential genetic basis, how a gene that affects a process of cerebellar neurodevelopment can generate such wide ranging abnormalities remains unclear.

## 2. The cerebellum and neurodevelopmental disorders

### 2.1. Cerebro-cerebellar interactions

As indicated above, NDD-associated behavioral abnormalities are consistent with cerebellar dysfunction. This is due to the extensive cerebellar connectivity (Figure 1A) with brain regions such as the prefrontal cortex, thalamus and ventral tegmental area (VTA). These broad networks underlie the cerebellar contribution to higher cognitive and affective processing, according to the specific region of the cerebellum that is activated (King et al., 2019).

However, functional impairment in NDDs is not limited to the cerebellum but involves the entire cerebellocortical circuit (Sathyanesan et al., 2019; Thabault et al., 2022). In schizophrenia, for example, there is reduced blood flow in the cerebello-thalamo-cortical circuit during a cognitive task (Daskalakis et al., 2005) and children with ASD display poor motor coordination that correlates with reduced cerebellar white matter fractional anisotropy (white matter integrity) (Dickinson et al., 2016). Cortico-cerebellar interactions are clearly demonstrated by fMRI and the functional connectivity between Right Crus 1 (RC1) and the infra-parietal lobule of the default mode network is disrupted in autistic patients (Stoodley et al., 2017). The infra-parietal lobule is involved in the imitation and interpretation of the gestures of other people, and an impairment of this network causes disruption of social development (Stoodley et al., 2017). The same functional connectivity occurs in mice, and when RC1 activity is experimentally inhibited, the mice display autistic-like behavior (Stoodley et al., 2017). The importance of RC1 is reinforced by its altered morphology in ASD patients, which correlates with an eye tracking abnormality (Laidi et al., 2017). Eye-tracking is thought to be involved in face recognition, thus an alteration in this function would account for decreased social interaction. Moreover, oculomotor impairment has recently been suggested as an early diagnostic feature for ASD (Laidi et al., 2017). The cerebellum also has direct projections to the VTA, which are involved in reward, particularly for social interaction, and cerebellar dysfunction in this pathway leads to abnormal social behavior, a classic NDD trait (Carta et al., 2019).

### 2.2. Cerebellar structural abnormalities in NDDs

In addition to connectivity errors, patients with NDDs often have reduced cerebellar volume (Daskalakis et al., 2005; Becker and Stoodley, 2013; Hampson and Blatt, 2015; Stoodley and Kuschner, 2016), and perinatal cerebellar injury forms the highest non-genetic risk for NDDs (Wang et al., 2014). Within the cerebellum, the cortex has a highly uniform network centered around Purkinje cells (PC), that receive excitatory input from climbing fiber axons of medullary inferior olive neurons and parallel fiber axons of cerebellar granular cells, in addition to modulation by local inhibitory interneurons. The PC is the sole cortical outflow, impacting on deep cerebellar neurons and thus downstream cerebral centers (Figure 1B). Patients with NDDs often show PC loss with different lobules being affected for each disorder (Daskalakis et al., 2005; Becker and Stoodley, 2013; Hampson and Blatt, 2015; Stoodley and Kuschner, 2016). The importance of PCs is confirmed in NDD mouse models in which gene mutations/deletions are limited to Purkinje cells, wherein PCs malfunction and there is associated ASD-like repetitive and social behaviors (Reith et al., 2013; Piochon et al., 2015; Thabault et al., 2022).

### 2.3. Cerebellar development

Cerebellar development is protracted extending from 30 days post-conception to the second post-natal year in humans and ∼E10 to P28 in mice (Figure 1C; Leto et al., 2016; van Essen et al., 2020) making it vulnerable to environmental change (Haldipur et al., 2011). The perinatal period is particularly vulnerable because it is the time of multiple changes in the cerebellum, including neurogenesis, neuronal migration and connectivity, all necessary for formation of the mature structure (Leto et al., 2016). But at the same time, major cerebellar projections to the thalamus, cortex and other regions are maturing. All these concurrent processes can explain the 36-fold increase in risk of ASD following perinatal cerebellar injury (Wang et al., 2014). The human perinatal period is equivalent to early post-natal stages in the mouse, in which the cerebellar cortical circuit is generated and refined (Leto et al., 2016). This period includes the genesis, migration and connectivity of granule cells (GCs) and their parallel fiber axons (PFs). Purkinje cells extend a large ramified dendritic tree to receive many PF synaptic inputs. They also receive multiple climbing fibers (CFs) from the brainstem inferior olive (Leto et al., 2016), which are refined to a monoinnervation by the end of the 3rd post-natal week. Lastly, basket, then stellate, inhibitory interneurons create negative feedback loops on PC activity (Ango et al., 2004). These processes occur in a defined order and if their appropriate timing is disturbed, PCs and their cortical circuit do not mature correctly (Letellier et al., 2009; Bailly et al., 2018), having modified activity that in turn will affect downstream cortical and subcortical areas and their associated cognitive and affective regulation. This hypothesis is supported by the existence of important changes in cerebrocerebellar interaction during maturation, which would indicate that disrupted cerebellar development could alter correct brain functioning (Moore et al., 2017): i.e., abnormal cerebellar development will not only alter its own function, but will also perturb the maturation of connected forebrain regions and their associated cognitive and affective processes. This impact that the development of one structure can have on another with which it is connected, was described by Wang et al. (2014) as *developmental diaschisis.*

Consistent with this hypothesis, a perinatal cerebellar lesion leads to a relative volume reduction of the contralateral prefrontal cortex (PFC; Limperopoulos et al., 2012). This relative size abnormality between the PFC and cerebellum also occurs in 3 to 9-year-old boys suffering from ASD (Carper, 2000; Sparks et al., 2002), thus supporting the importance of cerebellar development in NDDs and its developmental impact on the whole brain.

**FIGURE 2:**
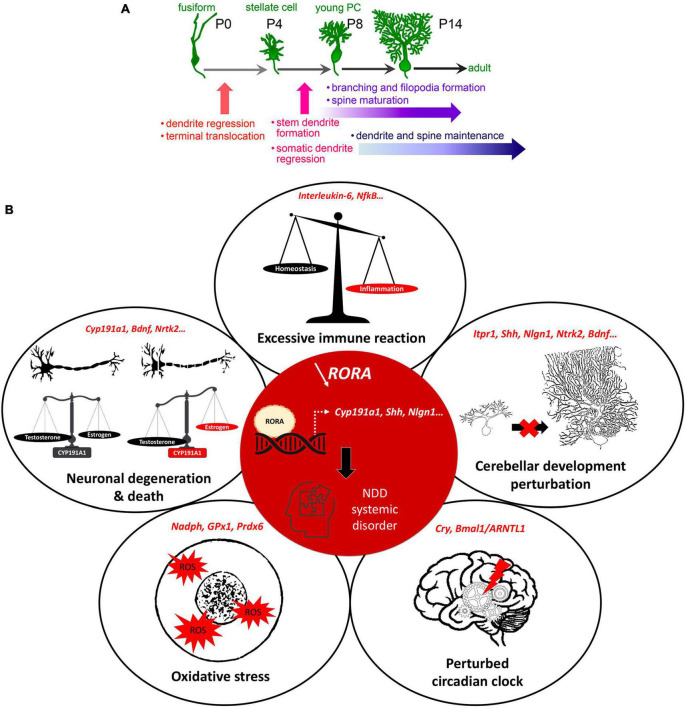
RORα regulates multiple genes and plays extensive roles in cerebellar development. **(A)** Key stages of PC development which are regulated by RORα. These are at all stages from embryonic development to adult maintenance. Reproduced from Takeo et al. (2015) with permission. **(B)** A schema showing the central role of RORα in multiple cellular processes, that are modified in NDDs. When RORα is reduced (central red circle), its regulation of gene transcription is altered. Here we include the known RORα target genes that are also involved in NDDs. The effects in red illustrate the induced abnormalities according to the direction of change: estrogen and PC development are reduced, circadian rhythms are perturbed, but inflammation and ROS are increased.

With so many interconnected processes taking place simultaneously during post-natal cerebellar development, multiple genes need to be expressed at a given time and in a given place. For example, sonic hedgehog (SHH) is secreted by PCs to stimulate granule cell precursor (GCP) division, thus shaping the cerebellum during pre and post-natal stages (Lewis et al., 2004). Synaptogenesis requires other genes such as neuroligins or shanks, mutations in which are known NDD risk factors (Parenti et al., 2020). Importantly, many genes expressed during cerebellar development, including 58 in PCs, are established risk-candidates for NDDs (Figure 1D; Clifford et al., 2019; Parenti et al., 2020). One such molecule is the Retinoic acid-related Orphan Receptor α (RORα), which is essential for PC development and maturation during the embryonic phase, for the refinement of their connections, and their maintenance throughout life (Figure 2A; Boukhtouche et al., 2006a; Chen et al., 2013; Takeo et al., 2015). Consequently, a lack of RORα will lead to PC malformation and death, and by interfering with PC secretion of SHH (Gold et al., 2003), it will reduce GC genesis, resulting in malformation of the whole cerebellar network.

Taken together, the high vulnerability of cerebellar development, including all developmental processes from gene expression to long-distance connectivity, combined with its close ties with cerebral structures such as the PFC, reinforce the role of this structure in the onset of NDDs.

## 3. RORα and neurodevelopmental disorders

Given the wide range of symptoms seen in NDDs, including impaired cognition, disruption of circadian clock, abnormal inflammatory episodes etc., it is difficult to imagine a single point of origin, including localized perinatal cerebellar injury. However, nuclear receptors, which are transcription factors activated by hormones, such as thyroid hormone, steroids and retinoic acid, have wide-ranging functions. Again, RORα, is of particular interest given its broad function *and* role in cerebellar development.

Retinoid-related Orphan Receptor alpha is a transcription factor with a classical structure including a ligand binding domain (LBD) and a DNA binding domain (DBD) that binds to ROR response elements (RORE) on the DNA (Becker-André et al., 1993). It exists in four isoforms (α1 to α4) differing by their N-terminal domain and by their level of expression in different tissues: RORα4 in leukocytes and skin, RORα2 and 3 in the testes and RORα1 and 4 in the brain (Matsui et al., 1995). Within the brain, RORα is widely expressed in the cerebral cortex, thalamus, hypothalamus and cerebellum (Ino, 2004). Consistent with being a transcription factor, RORα regulates a very large number of genes which results in RORα regulating many physiological processes including those that are disordered in NDDs (for review, see Cook et al., 2015). Moreover, studies on purified neurons show that RORα can bind to the promoter regions of over 2,500 genes (Hu et al., 2015), 438 of which are included in autism gene databases (Xu et al., 2012; Parenti et al., 2020).

More specifically for NDDs, RORα target genes are either confirmed NDD candidate genes (e.g., *ITPR1, NLGN1, NTRK2*) or regulate processes affected in NDDs (e.g., *CYP19A1, A2BP1, HSD17B10)* (Supplementary Table 1; Sarachana et al., 2011; Sarachana and Hu, 2013). For example, *CYP19A1* (aromatase) converts testosterone to estrogen, which upregulates RORα (Sarachana et al., 2011). Thus, in boys, less *RORA* expression and reduced aromatase will increase circulating testosterone, which in turn inhibits *RORA* expression (Sarachana et al., 2011), reinforcing the *RORA* deficiency. Moreover, less estrogen, which is neuroprotective, would exacerbate PC death, thus increasing the risk of developing autism (Janmaat et al., 2011; Hu et al., 2015) and explaining the sexual dimorphism of NDDs. This hypothesis is corroborated by post-mortem studies of autistic patients showing fewer PCs, which is more severe in males (Skefos et al., 2014), and also by the “extreme male brain theory” where patients with ASD traits have abnormal sex hormone balance, with higher testosterone (Greenberg et al., 2018). In addition, RORα also has anti-inflammatory functions through upregulating the antioxidant enzymes, glutathione peroxidase-1 and peroxiredoxin-6 (Boukhtouche et al., 2006b) that protect neurons and glia from the adverse effects of oxidative stress (Boukhtouche et al., 2006b; Jolly et al., 2012). RORα also regulates expression of the inflammatory cytokine interleukin 6 (IL6; Journiac et al., 2009). Thus, reduction of RORα function can underlie the greater inflammatory state found in NDD patients (Jiang et al., 2018). Lastly, RORα binds to Bmal1 and Cry and is a central part of the circadian cycle, a cycle known to be disrupted in ASD and schizophrenia patients (Jarvis et al., 2002; Hu et al., 2009). Taken together these data strongly suggest that RORα regulates cellular processes that are perturbed in NDDs (Figure 2B).

Further evidence suggesting that RORα is strongly implicated in NDDs comes from its expression in patient’s brains. RORα is reduced in the cerebellum and PFC of post-mortem ASD brains (Hu et al., 2015), and the *RORA* gene is hypermethylated (and therefore less expressed) in lymphoblastoid cells of these patients (Nguyen et al., 2010). Additionally, not only are *RORA* genetic variants associated with ASD (Sayad et al., 2017), but treatment of adult BTBR mice (an ASD model) with a synthetic RORα agonist improved repetitive behaviors by upregulating RORα target genes that are down-regulated in ASD (Wang et al., 2016). But the role of RORα is not confined to ASD; its expression is altered in schizophrenia (Devanna and Vernes, 2014) and many of its single nucleotide polymorphisms are found in ADHD (Miller et al., 2013; Liu et al., 2021). Furthermore, *RORA* missense variants can occur in either the DNA binding domain, conferring a dominant toxic effect, or in the ligand binding domain, which results in loss-of-function (Guissart et al., 2018). These data show how different mutations, and mutations at different loci in the *RORA* gene, can produce overlapping but distinct NDD phenotypes.

## 4. Discussion: Cerebellum, ROR, and NDDs

We have discussed separately the evidence for cerebellar and RORα involvement in neurodevelopmental disorders, but this does not automatically mean that the effects are linked. For example, there is greater PFC oxidative stress in schizophrenia and ASD (Rossignol and Frye, 2014; Maas et al., 2017), but there is also less PFC RORα expression in these disorders (Sarachana and Hu, 2013), which can directly explain the greater oxidative stress without involving the cerebellum.

However, RORα regulates multiple events during cerebellar development, and adult cerebellar maintenance, whose alteration can result in NDD-type dysfunction. This overlapping function reinforces the involvement of both the cerebellum *and* RORα in the development of these disorders. In the cerebellum, RORα is expressed in stellate and basket interneurons, but more particularly in Purkinje cells where it is required for their survival and growth (Boukhtouche et al., 2006a,b; Dusart and Flamant, 2012). As stated above, this effect on PC survival likely passes through RORα’s direct target genes, *CYP19A1* (aromatase) and *NTRK2* (the BDNF TrkB receptor), with subsequent reduction in activity of the trophic factors, estrogen and BDNF, respectively (Janmaat et al., 2011; Tsutsui et al., 2011). Moreover, in early post-natal development PCs secrete SHH to promote granule cell genesis and differentiation (Hamilton et al., 1996). However, RORα also directly regulates *SHH* expression (Gold et al., 2003). Thus, we propose that reduced RORα function explains not only poor PC maturation, but also their defective SHH secretion and subsequent impaired GC development (Leto et al., 2016).

The role of RORα continues during later stages of cerebellar development in particular the development of the Purkinje cell dendritic tree and cortical circuitry. In order to permit somatic polarization and growth of the dendritic tree, PCs regress their transient perisomatic dendrites; a process for which RORα is essential (Boukhtouche et al., 2006a). Subsequent dendritic expansion requires the genesis of GCs, with their PF axons and BDNF secretion (Altman, 1972; Tsutsui et al., 2011), both of which will be reduced by RORα deficiency through its regulation of *SHH* and *NTRKR2* (TrkB) expression (Sarachana and Hu, 2013). PF-PC synaptogenesis takes place on PC dendritic spines, whose formation depends on intracellular calcium regulation by IP3-induced Ca^2+^ release from the endoplasmic reticulum. RORα directly regulates *ITPR1* expression, thus its dysfunction will reduce ITPR1 transcription and hence, spine formation (Sugawara et al., 2017) altering the correct formation of the cerebellar cortical circuit. Indeed, in staggerer mice, which do not have any functional RORα, PCs do not develop dendritic spines, but this abnormality can be rescued by viral vector-induced *rora* expression, which induces the expression of RORα target genes (Iizuka et al., 2016). Furthermore, a second *RORA* target gene, *A2BP1*, codes for an RNA splicing enzyme, whose dysfunction in PCs causes abnormal splicing of *SCN8A* mRNA encoding the Nav1.6 sodium channel, a key mediator of Purkinje cell pace-making. Thus, although PCs appear normal, they have abnormal spiking activity (Gehman et al., 2012), which will impact upon cerebello-cerebral function. Finally in the 2nd−3rd post-natal weeks, PC activity becomes closely modulated by inhibitory molecular layer interneurons, stellate (SC) and basket cells (BCs), which are the last to form in the immature cerebellar cortex (Leto et al., 2016). The post-synaptic cell adhesion protein, Neuroligin 1, is required for normal GABAergic input to PCs through synaptogenesis at PF-SCs and at the BC-PC axon synaptic complex (Nozawa et al., 2018). Importantly, RORα upregulates the *NLGN1* gene (Sarachana and Hu, 2013) thus contributing to GABAergic regulation of cerebellar cortical activity. Therefore, when RORα is deficient, there will be an alteration in excitatory/inhibitory balance in PC afferents. In addition to these key functions during development, RORα is required throughout life to maintain PC dendrites (Chen et al., 2013) and survival, for example ITPR1 deficiency causes the adult-onset spinocerebellar ataxia 15 with its associated PC loss (Sugawara et al., 2017).

Although we present multiple roles for RORα in cerebellar development and function that are likely mechanisms underlying NDDs, we do not claim that it is *the* cause. There is often an environmental component to NDDs, which is consistent with the prolonged period of cerebellar development, and this will be independent from RORα dysfunction. Moreover, the genetics of NDDs is vast, with numerous small missense mutations in many “risk” genes (Parenti et al., 2020), which often have to occur in combination to cause an NDD phenotype. Therefore, combining RORα ’s role in regulating circadian rhythms, oxidative stress and inflammation (Jarvis et al., 2002; Boukhtouche et al., 2006b; Hu et al., 2015), all key comorbidities in NDDs, with its role in cerebellar development, we hypothesize that RORα has a major causative role in NDD pathophysiology. Further investigation to confirm this possibility is essential because it points to an NDD-risk gene that has appropriate widespread actions, and which can be replaced therapeutically to upregulate the abnormal low expression of *RORA* target genes, thus improving abnormal NDD-linked behaviors.

## Data availability statement

The original contributions presented in this study are included in the article/Supplementary material, further inquiries can be directed to the corresponding author.

## Author contributions

SR wrote the original draft. SR and RS edited and finalized the manuscript. Both authors contributed to the article and approved the submitted version.
